# Supplementary material for: Assessing the Intergenerational Linkage between Short Maternal Stature and Under-Five Stunting and Wasting in Bangladesh
Source: Nutrients. 2019 Aug 7;11(8):1818. doi: 10.3390/nu11081818 (PMC6722712; doi:10.3390/nu11081818)
Supplement: Supplementary file 1 [file nutrients-11-01818-s001.pdf]

Supplementary Appendix

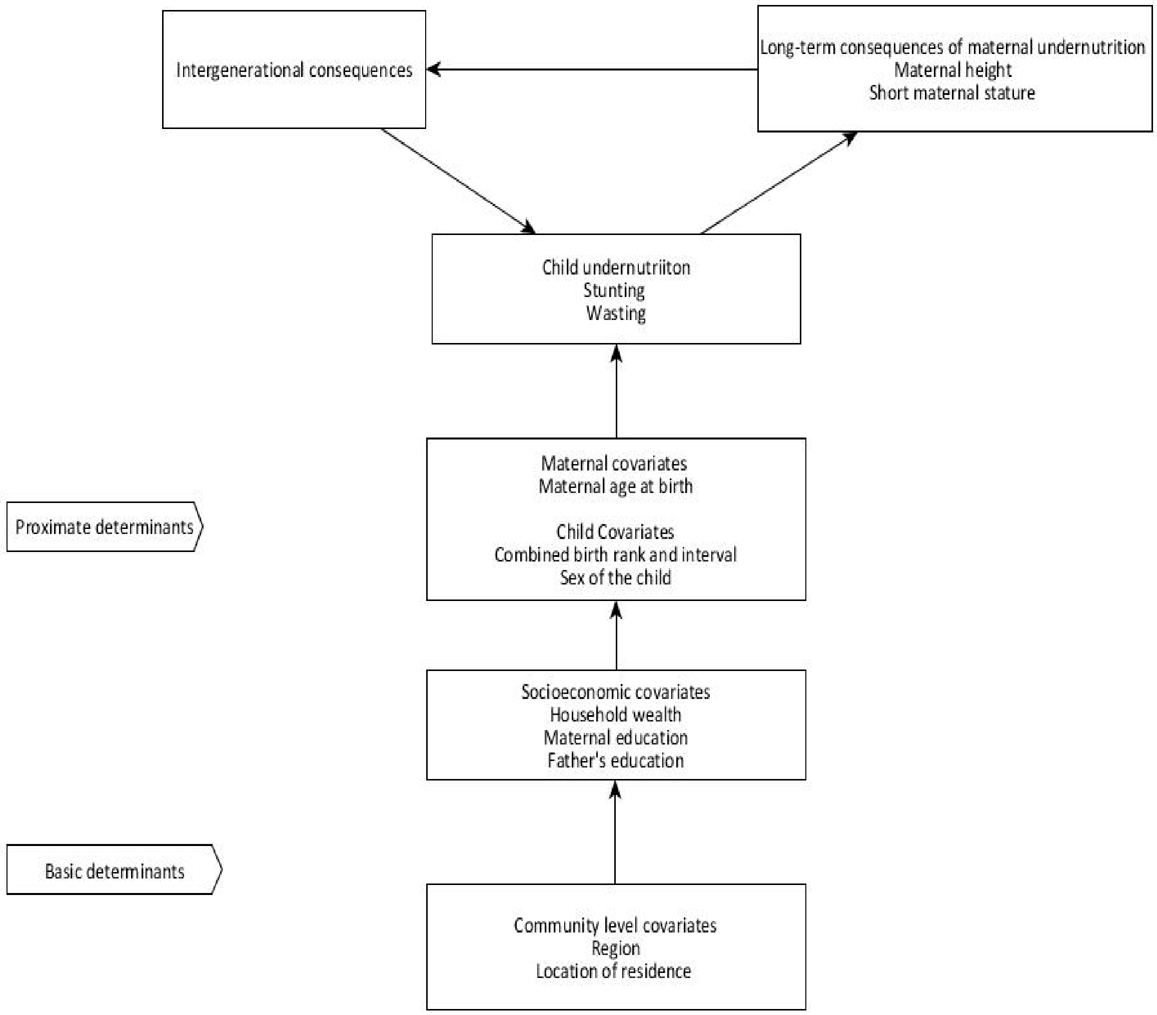

Figure S1 Conceptual framework for assessing association between maternal height and offspring stunting and wasting. (adapted from UNICEF 2013)

Table S1 Frequency distribution (weighted) of the covariates across the maternal height categories in child anthropometry datasets

	Maternal height categories, cm			
	≥155.0	154.9-150.0	149.9-145.0	<145.0
**Covariates**	**N=5634**	**N=8748**	**N=7779**	**N=3477**
**Maternal covariates**				
<20	1490(27.0)	2388(28.2)	2185(28.9)	1022(29.6)
20-24	1894(33.7)	2943(33.5)	2615(33.4)	1168(33.6)
25-29	1314(22.7)	1956(22.0)	1696(22.1)	725(21.5)
≥30	936(16.6)	1461(16.3)	1283(15.7)	562(15.3)
Educational level				
No education	1081(20.2)	1890(22.2)	1981(26.3)	1075(32.2)
Primary	1491(27.2)	2574(29.7)	2424(30.7)	1251(35.6)
Secondary	2397(42.6)	3495(40.1)	2879(37.2)	1012(28.9)
Higher	665(9.9)	788(7.9)	494(5.7)	139(3.4)
Occupation				
Not working	4645(82.0)	7109(79.8)	6356(81.0)	2805(79.9)
Working	989(18.0)	1638(20.2)	1423(19.0)	672(20.1)
**Child Covariates**				
Birth Order				
First	2083(36.5)	3163(35.7)	2592(33.8)	1171(32.8)
Second	1584(27.6)	2330(27.1)	2099(27.1)	911(26.4)
Third	927(17.5)	1425(16.4)	1409(17.9)	604(18.4)
Fourth	485(8.8)	849(9.9)	760(9.6)	359(10.6)
≥ Fifth	555(9.5)	981(11.0)	919(11.5)	432(11.9)
Birth Interval				
First child	2083(36.5)	3163(35.7)	2592(33.8)	1171(32.8)
≤23 months	448(7.8)	711(7.7)	668(8.2)	329(9.5)
24-47 months	1329(23.5)	2182(25.7)	2052(26.8)	983(28.7)
≥48 months	1774(32.1)	2692(30.9)	2467(31.2)	994(29.1)
Sex of the child				
Male	2853(50.9)	4457(50.7)	3999(51.3)	1753(50.4)
Female	2781(49.1)	4291(49.3)	3780(48.7)	1724(49.6)
**Household covariates**				
Wealth Quintile				
First, poorest	1095(17.5)	1775(18.2)	1829(20.9)	954(24.9)
Second	887(17.1)	1597(19.4)	1484(20.5)	754(25.0)
Third	982(18.8)	1696(20.5)	1497(20.0)	725(20.0)
Fourth	1164(21.4)	1684(19.8)	1558(20.3)	607(18.0)
Fifth, richest	1506(25.2)	1996(22.1)	1411(18.2)	437(12.2)
Father's Education				
No education	1482(27.8)	2490(29.9)	2539(33.6)	1355(41.5)
Primary	1431(26.2)	2500(28.8)	2311(29.7)	1122(30.5)
Secondary	1714(30.0)	2534(28.6)	2080(26.5)	772(22.2)
Higher	1007(16.1)	1224(12.7)	849(10.2)	228(5.9)
Location of Residence				
urban	1874(23.1)	2762(22.6)	2392(21.7)	1043(20.6)

rural	3760(77.0)	5986(77.4)	5387(78.3)	2434(79.4)
Region				
Barisal	622(5.6)	1033(6.0)	941(6.0)	383(5.5)
Chittagong	1134(22.4)	1811(22.3)	1612(22.5)	624(18.8)
Dhaka	992(30.5)	1614(31.8)	1518(32.6)	731(35.8)
Khulna	853(11.9)	1059(9.8)	824(8.1)	316(6.8)
Rajshahi	869(16.3)	1243(15.7)	1083(15.7)	546(17.4)
Sylhet	703(8.7)	1191(9.5)	1093(10.1)	527(10.1)
Rangpur	461(4.6)	797(4.9)	708(4.9)	350(5.7)
Time (year)				
2004	1179(21.1)	1967(22.5)	1876(23.6)	896(26.4)
2007	1074(18.1)	1778(19.9)	1586(20.6)	744(20.7)
2011	1727(31.1)	2608(29.6)	2279(29.1)	972(27.5)
2014	1654(29.7)	2395(28.1)	2038(26.7)	865(25.3)

Table S2 Association of maternal height (cm) with severe stunting of the under-five children adjusted for other covariates, unadjusted and adjusted relative risk with 95% confidence interval

	Severely stunted* under-five children					
	Unadjusted		Adjusted model 1[a]		Adjusted model 2[b]	
Covariates	RR (95%CI)	P value	RR (95%CI)	P value	RR (95%CI)	P value
**Maternal covariates**						
Maternal height per 1-cm increase	0.934(0.928-0.941)	<0.001	0.941(0.935-0.946)	<0.001		
Maternal height, cm						
≥155.0 cm (tall)	1 [Reference]				1 [Reference]	
154.9-150.0 cm	1.55(1.38,1.75)				1.46(1.30,1.64)	
149.9-145.0 cm	2.38(2.12,2.67)				2.12(1.90,2.37)	
<145.0 cm (short)	3.61(3.21,4.06)	<0.001			2.97(2.65,3.33)	<0.001
Maternal Age at birth, y						
<20	1 [Reference]		1 [Reference]		1 [Reference]	
20-24	0.92(0.85,1.00)		0.84(0.76,0.92)		0.84(0.77,0.93)	
25-29	0.90(0.81,0.99)		0.71(0.63,0.81)		0.72(0.63,0.81)	
≥30	1.13(1.03,1.24)	<0.001	0.72(0.63,0.83)	<0.001	0.72(0.62,0.83)	<0.001
Maternal Educational level						
No education	1 [Reference]		1 [Reference]		1 [Reference]	
Primary	0.80(0.74,0.86)		0.96(0.89,1.04)		0.97(0.89,1.04)	
Secondary	0.46(0.42,0.50)		0.80(0.72,0.89)		0.81(0.73-0.90)	
Higher	0.23(0.18,0.29)	<0.001	0.73(0.56,0.95)	<0.001	0.74(0.5660.96)	<0.001
Mother's Occupation						
Not working	1 [Reference]					
Working	1.07(0.99,1.16)	0.103				
**Child Covariates**						
child age category, mo						
0-11	1 [Reference]		1 [Reference]		1 [Reference]	
12-23	2.46(2.16,2.81)		2.48(2.18,2.82)		2.48(2.18,2.82)	
24-35	2.72(2.39,3.09)		2.64(2.32-3.00)		2.64(2.32,3.00)	
36-47	2.84(2.50,.23)		2.72(2.40-3.08)		2.70(2.38,3.07)	
48-59	2.25(1.96,2.58)	<0.001	2.07(1.81-2.37)	<0.001	2.09(1.83,2.39)	<0.001
Birth Order						
First	1 [Reference]		1 [Reference]		1 [Reference]	
Second	1.11(1.01,1.21)		1.11(1.01,1.23)		1.11(1.00,1.23)	
Third	1.22(1.11,1.35)		1.17(1.04,1.32)		1.16(1.03,1.31)	
Fourth	1.56(1.39,1.75)		1.40(1.20,1.63)		1.41(1.21,1.63)	
≥ Fifth	1.96(1.78,2.15)	<0.001	1.56(1.34,1.82)	<0.001	1.55(1.33,1.81)	<0.001
Birth Interval						
First child	1 [Reference]					
≤23 months	1.76(1.58,1.95)					

24-47 months	1.61(1.48,1.75)					
≥48 months	1.02(0.93,1.11)	<0.001				
Sex of the child						
Male	1 [Reference]					
Female	0.97(0.91,1.03)	0.394				
Household covariates						
Wealth Quintile						
First, poorest	1 [Reference]		1 [Reference]		1 [Reference]	
Second	0.89(0.82,0.97)		0.96(0.89,1.04)		0.96(0.89,1.04)	
Third	0.72(0.66,0.78)		0.86(0.78,0.94)		0.87(0.79,0.95)	
Fourth	0.52(0.46,0.58)		0.71(0.64,0.80)		0.72(0.63,0.80)	
Fifth, richest	0.29(0.26,0.33)	<0.001	0.51(0.44,0.58)	<0.001	0.52(0.45,0.59)	<0.001
Father's Education						
No education	1 [Reference]		1 [Reference]			
Primary	0.78(0.73,0.842)		0.94(0.87,1.01)			
Secondary	0.53(0.49,0.581)		0.85(0.77,0.94)			
Higher	0.27(0.23,0.320)	<0.001	0.63(0.52,0.77)	<0.001		
Location of Residence						
urban	1 [Reference]					
rural	1.29(1.20,1.397)	<0.001				
Region						
Barisal	1 [Reference]		1 [Reference]		1 [Reference]	
Chittagong	0.96(0.86,1.060)		1.03(0.94,1.14)		1.05(0.95,1.16)	
Dhaka	0.84(0.75,0.933)		0.86(0.78,0.96)		0.87(0.78,0.96)	
Khulna	0.55(0.48,0.632)		0.64(0.56,0.74)		0.65(0.57,0.74)	
Rajshahi	0.73(0.64,0.823)		0.67(0.59,0.75)		0.67(0.60,0.76)	
Sylhet	0.92(0.82,1.035)		0.92(0.820,1.02)		0.92(0.83,1.03)	
Rangpur	1.14(1.00,1.283)	<0.001	1.32(1.16,1.49)	<0.001	1.33(1.17,1.50)	<0.001
Time (year)						
2004	1 [Reference]		1 [Reference]		1 [Reference]	
2007	0.78(0.71,0.849)		0.85(0.78,0.93)		0.86(0.79,0.93)	
2011	0.70(0.650,0.762)		0.79(0.73,0.86)		0.79(0.73,0.86)	
2014	0.53(0.48,0.589)	<0.001	0.65(0.58,0.72)	<0.001	0.65(0.59,0.72)	<0.001
Recall	1.001(0.999-1.003)	0.092				

Note: Abbreviations: * Severely Stunted: height for age Z score < -3 SD; RR, Relative Risk; CI, Confidence Interval; [a] Adjusted model 1: maternal height in cm is considered as the continuous variables while adjusting for other covariates; [b] Adjusted model 2: maternal height in cm is considered as the categorical variables while adjusting for other covariates

Table S3 Association between stunting and maternal height (cm) adjusted for significant interaction between maternal height and other covariates, unadjusted and adjusted relative risk with 95% confidence interval

	Stunted* under-five children					
	Unadjusted		Adjusted model 1		Adjusted model 2	
**Covariates**	**RR (95%CI)**	**P Value**	**RR (95%CI)**	**P Value**	**RR (95%CI)**	**P Value**
**Maternal covariates**						
Maternal height per 1-cm increase	0.954 (0.951,0.958)	<0.001	0.960 (0.957,0.963)	0.001		
Maternal height, cm						
≥155.0 cm (tall)	1[Reference]				1[Reference]	
154.9-150.0 cm	1.45(1.36,1.54)				1.90(1.58,2.29)	
149.9-145.0 cm	1.86(1.75,1.98)				2.42(2.01,2.91)	
<145.0 cm (short)	2.36(1.22,2.51)	<0.001			3.52(2.89,4.29)	<0.001
Maternal Age at birth, y						
<20	1 [Reference]		1 [Reference]		1[Reference]	
20-24	0.96(0.92,1.00)		0.93(0.89,0.98)		0.94(0.89,0.98)	
25-29	0.89(0.85, 0.94)		0.82(0.77,0.87)		0.82(0.77,0.87)	
≥30	1.02(0.97,1.07)		0.81(0.75,0.87)	<0.001	0.82(0.76,0.88)	<0.001
Maternal Educational level						
No education	1 [Reference]		1 [Reference]		1[Reference]	
Primary	0.91(0.88,0.95)		1.02(0.977,1.06)		1.02(0.98,1.06)	
Secondary	0.67(0.64,0.70)		0.94(0.89,0.99)		0.94(0.90,0.99)	
Higher	0.39(0.35,0.44)		0.81(0.72,0.92)	<0.001	0.82(0.75,0.93)	<0.001
Mother's Occupation						
Not working	1 [Reference]					
Working	1.09(1.05,1.14)					
**Child Covariates**						
child age category, mo						
0-11	1 [Reference]		1 [Reference]		1[Reference]	
12-23	2.21(2.05,2.38)		2.21(2.05,2.37)		2.21(2.05,2.37)	
24-35	2.37(2.20,2.55)		2.34(2.81,2.52)		2.35(2.19,2.52)	
36-47	2.42(2.25,2.60)		2.36(2.20,2.54)		2.36(2.20,2.53)	
48-59	2.12(1.97,2.29)	<0.001	2.03(1.89,2.18)	<0.001	2.04(1.90,2.20)	<0.001
Birth Order						
First	1 [Reference]		1 [Reference]		1 [Reference]	
Second	1.06(1.01,1.11)		1.06(1.01,1.11)		1.18(1.02,1.36)	
Third	1.12(1.06,1.18)		1.08(1.02,1.15)		1.30(1.11,1.52)	
Fourth	1.27(1.20,1.34)		1.20(1.11,1.29)		1.46(1.23,1.74)	
≥ Fifth	1.46(1.39,1.54)	<0.001	1.30(1.20,1.40)	<0.001	1.74(1.49,2.04)	<0.001
Birth Interval						
First child	1 [Reference]					
≤23 months	1.37(1.29,1.45)					
24-47 months	1.30(1.25,1.36)					
≥48 months	1.10(0.97,1.06)	<0.001				
Sex of the child						
Male	1 [Reference]					
Female	1.00(9.67,1.04)	0.999				

Household covariates						
Wealth Quintile						
Fifth (wealthiest)	1 [Reference]		1 [Reference]		1 [Reference]	
Fourth	1.47(1.37,1.58)		1.26(1.18,1.35)		1.48(1.22,1.79)	
Third	1.72(1.62,1.84)		1.34(1.26,1.44)		1.62(1.34,1.96)	
Second	1.99(1.87,2.11)		1.46(1.37,1.56)		1.78(1.48,2.13)	
First (poorest)	2.09(1.97,2.22)	<0.001	1.51(1.41,1.61)	<0.001	1.94(1.62,2.31)	<0.001
Father's Education						
No education	1 [Reference]		1 [Reference]		1 [Reference]	
Primary	0.88(0.85,0.92)		0.98(0.94,1.02)		0.98(0.94,1.02)	
Secondary	0.69(0.66,0.72)		0.90(0.86,0.95)		0.90(0.86,0.95)	
Higher	0.45(0.42,0.49)	<0.001	0.76(0.70,0.83)	<0.001	0.76(0.69,0.83)	<0.001
Location of Residence						
urban	1 [Reference]		1 [Reference]		1 [Reference]	
rural	1.22(1.17,1.27)	<0.001	1.08(1.04,1.13)	<0.001	1.08(1.04,1.13)	<0.001
Region						
Barisal	1 [Reference]		1 [Reference]		1 [Reference]	
Chittagong	0.95(0.90,0.998)		1.01(0.96,1.06)		1.02(0.97,1.07)	
Dhaka	0.92(0.88,0.972)		0.95(0.90,1.01)		0.96(0.91,1.01)	
Khulna	0.75(0.70,0.801)		0.84(0.79,0.89)		0.84(0.79,0.90)	
Rajshahi	0.86(0.81,0.914)		0.83(0.78,0.88)		0.83(0.79,0.88)	
Sylhet	0.93(0.88,0.995)		0.93(0.88,0.99)		0.94(0.88,0.99)	
Rangpur	1.07(1.00,0.140)	<0.001	1.15(1.07,1.22)	<0.001	1.15(1.07,1.22)	<0.001
Time (year)						
2004	1 [Reference]		1 [Reference]		1 [Reference]	
2007	0.89(0.847,0.928)		0.93(0.90,0.98)		0.94(0.90,0.92)	
2011	0.81(0.780,0.848)		0.88(0.84,0.92)		0.89(0.85,0.93)	
2014	0.72(0.687,0.760)		0.82(0.78,0.89)	<0.001	0.83(0.79,0.87)	<0.001
Recall	1.003(1.002-1.003)					
Maternal height category (cm)#Birth order						
≥155.0#First child					1 [Reference]	
154.9-150.0 cm #Second					0.91(0.77,1.07)	
154.9-150.0 cm #Third					0.75(0.63,0.90)	
154.9-150.0 cm #Fourth					0.78(0.64,0.95)	
154.9-150.0 cm #≥Fifth					0.71(0.60,0.84)	
149.9-145.0 cm #Second					0.89(0.76,1.04)	
149.9-145.0 cm #Third					0.85(0.72,1.01)	
149.9-145.0 cm #Fourth					0.81(0.70,0.98)	
149.9-145.0 cm #≥Fifth					0.71(0.60,0.84)	
<145.0 cm #Second					0.84(0.71,0.99)	
<145.0 cm #Third					0.81(0.67,0.97)	
<145.0 cm #Fourth					0.78(0.65,0.95)	
<145.0 cm #≥Fifth					0.69(0.58,0.82)	0.004
Maternal height category (cm)# wealth quintile						
≥155 cm # 5th quintile (Wealthiest)					1 [Reference]	
154.9-150.0 cm # 4th quintile					0.87(0.70,1.09)	

154.9-150.0 cm # 3rd quintile	0.80(0.64,1.00)	
154.9-150.0 cm # 2nd quintile	0.84(0.69,1.04)	
154.9-150.0 cm # 1st quintile (Poorest)	0.82(0.67,1.00)	
149.9-145.0 cm # 4th quintile	0.80(0.64,1.01)	
149.9-145.0 cm # 3rd quintile	0.82(0.66,1.02)	
149.9-145.0 cm # 2nd quintile	0.80(0.65,0.98)	
149.9-145.0 cm # 1st quintile (Poorest)	0.75(0.62,0.92)	
<145.0 cm # 4th quintile	0.75(0.60,0.95)	
<145.0 cm # 3rd quintile	0.72(0.57,0.90)	
<145.0 cm # 2nd quintile	0.67(0.54,0.84)	
<145.0 cm # 1st quintile (Poorest)	0.59(00.48,0.73)	<0.001

Note: Abbreviations: * Stunted: height for age Z score < -2 SD; RR, Relative Risk; CI, Confidence Interval [a]Adjusted model 1: maternal height in cm is considered as the continuous variables while adjusting for other covariates; [b] Adjusted model 2: maternal height in cm is considered as the categorical variables while adjusting for other covariates

Table S4 Association of maternal height (cm) with severe wasting of the under-five children adjusted for other covariates, unadjusted and adjusted relative risk with 95% confidence interval

	Severely wasted+ under-five children					
	**Unadjusted**		**Adjusted model 1[a]**		**Adjusted model 2[b]**	
**Covariates**	**RR (95%CI)**	**P Value**	**RR (95%CI)**	**P Value**	**RR (95%CI)**	**P Value**
**Maternal covariates**						
Maternal height per 1-cm increase	0.984 (0.971,0.997)	0.016	0.984 (0.971,0.997)	0.020		
Maternal height, cm						
≥155.0 cm (tall)	1 [Reference]				1 [Reference]	
154.9-150.0 cm	1.20(1.00,1.495)				1.20(0.96,1.49)	
149.9-145.0 cm	1.23(0.98,1.542)				1.22(0.98,1.54)	
<145.0 cm (short)	1.44(1.12,1.851)	0.039			1.43(1.11,1.83)	0.049
Maternal Age at birth, y						
<20	1 [Reference]					
20-24	0.88(0.72,1.076)					
25-29	1.13(0.91,1.402)					
≥30	1.12(0.90,1.402)	0.073				
Maternal Educational level						
No education	1 [Reference]					
Primary	0.98(0.80,1.183)					
Secondary	0.83(0.68,1.012)					
Higher	0.70(0.48,1.024)	0.085				
Mother's Occupation						
Not working	1 [Reference]					
Working	0.90(0.73,1.102)	0.304				
**Child Covariates**						
Child age category, mo						
0-11	1 [Reference]		1 [Reference]		1 [Reference]	
12-23	0.85(0.69,1.06)		0.86(0.70,1.06)		0.86(0.70,1.06)	
24-35	0.60(0.48,0.76)		0.60(0.48,0.75)		0.60(0.48,0.75)	
36-47	0.52(0.41,0.67)		0.51(0.40,0.65)		0.51(0.40,0.65)	
48-59	0.47(0.36,0.61)	<0.001	0.46(0.35,0.59)	<0.001	0.46(0.36,0.60)	<0.001
Birth Order						
First	1 [Reference]		1 [Reference]		1 [Reference]	
Second	0.96(0.78,1.18)		0.98(0.79,1.20)		0.98(0.79,1.20)	
Third	1.09(0.87,1.37)		1.11(0.89,1.40)		1.11(0.89,1.40)	
Fourth	1.36(1.06,1.73)		1.41(1.10,1.80)		1.41(1.10,1.80)	
≥ Fifth	1.33(1.05,1.68)	0.015	1.41(1.11,1.78)	0.003	1.41(1.11,1.78)	0.003
Birth Interval						
First child	1 [Reference]					
≤23 months	1.10(0.83,1.47)					
24-47 months	1.21(0.99,1.48)					
≥48 months	1.04(0.86,1.25)	0.269				

Sex of the child						
Male						
Female	0.92(0.86,0.98)	0.003	0.79(0.68,0.93)	0.003	0.79(0.68,0.93)	0.003
Household covariates						
Wealth Quintile						
First, poorest	1 [Reference]					
Second	0.83(0.67,1.04)					
Third	0.84(0.68,1.05)					
Fourth	0.75(0.60,0.94)					
Fifth, richest	0.68(0.53,0.88)	0.027				
Father's Education						
No education	1 [Reference]					
Primary	0.98(0.82,1.18)					
Secondary	0.84(0.68,1.02)					
Higher	0.63(0.47,0.85)	0.010				
Location of Residence						
urban	1 [Reference]					
rural	1.07(0.89,1.27)	0.485				
Region						
Barisal	1 [Reference]					
Chittagong	1.24(0.95,1.62)					
Dhaka	0.96(0.74,1.26)					
Khulna	1.18(0.88,1.57)					
Rajshahi	1.11(0.84,1.47)					
Sylhet	1.22(0.88,1.69)					
Rangpur	1.03(0.74,1.44)	0.318				
Time (year)						
2004	1 [Reference]		1 [Reference]		1 [Reference]	
2007	0.89(0.71,1.11)		0.89(0.71,1.12)		0.90(0.71,1.13)	
2011	1.15(0.94,1.39)		1.20(0.99,1.46)		1.20(0.99,1.46)	
2014	0.92(0.73,1.15)	0.001	0.96(0.77,1.20)	0.029	0.96(0.77,1.12)	0.028
Recall	0.996(0.994-0.999)	0.014				

Note: Abbreviations: [+]Severely Wasted: weight for height Z score <-2SD; RR, Relative Risk; CI, Confidence Interval [a] Adjusted model 1: maternal height in cm is considered as the continuous variables while adjusting for other covariates; [b] Adjusted model 2: maternal height in cm is considered as the categorical variables while adjusting for other covariates.
